# Therapeutic Effect of a Novel Phosphatidylinositol-3-Kinase δ Inhibitor in Experimental Epidermolysis Bullosa Acquisita

**DOI:** 10.3389/fimmu.2018.01558

**Published:** 2018-07-12

**Authors:** Hiroshi Koga, Anika Kasprick, Rosa López, Mariona Aulí, Mercè Pont, Núria Godessart, Detlef Zillikens, Katja Bieber, Ralf J. Ludwig, Cristina Balagué

**Affiliations:** ^1^Lübeck Institute of Experimental Dermatology, University of Lübeck, Lübeck, Germany; ^2^Skin Biology and Pharmacology, Almirall R&D, Barcelona, Spain; ^3^Preclinical Safety and Toxicology, Almirall R&D, Barcelona, Spain; ^4^Department of Dermatology University of Lübeck, Lübeck, Germany

**Keywords:** phosphatidylinositol-3-kinase, skin, autoimmunity, animal models, treatment, preclinical testing, pemphigoid, epidermolysis bullosa acquisita

## Abstract

Epidermolysis bullosa acquisita (EBA) is a rare, but prototypical, organ-specific autoimmune disease, characterized and caused by autoantibodies against type VII collagen (COL7). Mucocutaneous inflammation, blistering, and scarring are the clinical hallmarks of the disease. Treatment of EBA is difficult and mainly relies on general immunosuppression. Hence, novel treatment options are urgently needed. The phosphatidylinositol-3-kinase (PI3K) pathway is a putative target for the treatment of inflammatory diseases, including EBA. We recently discovered LAS191954, an orally available, selective PI3Kδ inhibitor. PI3Kδ has been shown to be involved in B cell and neutrophil cellular functions. Both cell types critically contribute to EBA pathogenesis, rendering LAS191954 a potential drug candidate for EBA treatment. We, here, demonstrate that LAS191954, when administered chronically, dose-dependently improved the clinical phenotype of mice harboring widespread skin lesions secondary to immunization-induced EBA. Direct comparison with high-dose corticosteroid treatment indicated superiority of LAS191954. Interestingly, levels of circulating autoantibodies were unaltered in all groups, indicating a mode of action independent of the inhibition of B cell function. In line with this, LAS191954 also hindered disease progression in antibody transfer-induced EBA, where disease develops dependent on myeloid, but independent of B cells. We further show that, *in vitro*, LAS191954 dose-dependently impaired activation of human myeloid cells by relevant disease stimuli. Specifically, immune complex-mediated and C5a-mediated ROS release were inhibited in a PI3Kδ-dependent manner. Accordingly, LAS191954 also modulated the dermal–epidermal separation induced *in vitro* by co-incubation of immune complexes with polymorph nuclear cells, thus pointing to an important role of PI3Kδ in EBA effector functions. Altogether, these results suggest a new potential mechanism for the treatment of EBA and potentially also other autoimmune bullous diseases.

## Introduction

Pemphigoid diseases comprise a group of autoimmune disorders with a high, and so far, unmet medical need. They are clinically characterized by chronic (muco)-cutaneous inflammation and subepidermal blistering, and are caused by autoantibodies targeting structural proteins of the dermal–epidermal junction (1–4). Pemphigoid diseases pose an immense burden on the affected patients, including an increased mortality (5), and are difficult to treat. For example, bullous pemphigoid, characterized by autoantibodies targeting type XVII collagen (COL17) respond well to corticosteroid treatment, but after stopping treatment, the disease frequently relapses (6). This requires prolonged, and often systemic, corticosteroid treatment. In contrast, the pemphigoid disease epidermolysis bullosa acquisita (EBA), characterized by autoantibodies against type VII collagen (COL7), is notoriously refractory to many treatments. Even after prolonged immunosuppressive treatment, many patients fail to reach a clinical remission (7, 8). Hence, there is a yet high unmet medical need for the development of novel treatments for pemphigoid diseases, especially EBA (3).

Animal models of EBA have provided detailed insights into the disease pathogenesis (9, 10). During the induction phase of the disease, COL7-autoreactive plasma cells are generated in a CD4-dependent fashion (11), which lead to the production of antibodies to COL7. In the effector phase, these autoantibodies bind to their cognate target antigen at the dermal–epidermal junction and trigger a cascade of events that include the activation of the complement network, recruitment of myeloid cells (12, 13), and engagement of Fcγ receptors (14). This in turn leads to the activation of signal transduction pathways downstream of Syk (15) and Src kinases (16), which also include PI3Kβ (17, 18), resulting in the release of potent inflammatory mediators, such as cytokines, reactive oxygen species, and proteases, which combined are instrumental for lesion formation.

The PI3Kδ-dependent pathways are activated in various inflammatory and cancerous conditions, and pharmacological inhibition or genetic inactivation of this pathway has demonstrated efficacy in several preclinical models of inflammation as well as in lymphoma patients (19). We hypothesized that the role of PI3Kδ could be critical in the context of the postulated EBA pathogenesis by directly impacting in the function of critical cellular players. On the one hand, genetic and pharmacological studies with specific PI3Kδ inhibitors have shown that antibody responses to T cell-dependent antigens as well as the production of autoantibodies in some models are PI3Kδ dependent (20–22). In addition, PI3Kδ is required for distinct neutrophil functions *in vitro* and *in vivo* such as neutrophil directional migration and degranulation in response to distinct receptor activations (23–25). Hence, pharmacological targeting of the PI3Kδ pathway could block two crucial pathways in EBA pathogenesis, namely autoantibody formation and production, and FcγR-mediated myeloid cell activation.

Based on the above considerations, we here evaluated the effect of LAS191954, a recently described novel selective PI3Kδ inhibitor (26), on clinical aspects of distinct experimental EBA mouse models. Our results indicate that the compound can reverse established immunization-induced disease and prevent disease induced by anti-COL7 antibody transfer. Further assessment of the mechanism of action suggests that these effects are greatly contributed by the inhibition of myeloid cell function in a predominantly PI3Kδ-dependent manner.

## Materials and Methods

### Experiments With Human Biomaterials

Foreskin and blood collections from healthy volunteers were performed after written informed consent was obtained. All experiments with human samples were approved by the ethical committee of the Medical Faculty of the University of Lübeck and were performed in accordance with the Declaration of Helsinki.

### Laboratory Animals

C57Bl/6 (B6) and B6.SJL-H2s (B6.s) mice were obtained from colonies held at the animal facility at the University of Lübeck. Mice were housed under specific pathogen-free conditions and provided standard mouse chow and acidified drinking water *ad libitum*. Mice aged 6–10 weeks were used for the experiments. All clinical examinations, biopsies, and bleedings were performed under anesthesia with i.p. administration of a mixture of ketamine (100 µg/g) and xylazine (15 µg/g). Evaluation of skin lesions was performed as described (10). Animal experiments were approved by local authorities of the Animal Care and Use Committee (Kiel, Germany) and performed by certified personnel. For KLH immunization studies, male Crl:CD1 (ICR) mice were purchased from Charles River. Female MRL/MpJ-Faslpr/J mice were purchased from The Jackson Laboratory (Bar Harbor, ME, USA). Animals were housed in polycarbonate cages, with free access to water and non-purified stock diet and allowed to condition for 2 weeks in their new environment at 22 ± 2°C, 40–70% relative humidity, and 12 h:12 h light:dark cycles. All animal care and experimental procedures followed the European Community Directive 86/609/CEE and the Autonomous Catalan law (Decret 214/1997) for the use of laboratory animals and were approved by the Almirall Animal Experimentation Ethical Committee.

### Chemicals

LAS191954 was synthesized as previously described (26). For *in vivo per os* administration, LAS191954 was suspended in 0.5% methylcellulose, 0.1% Tween80. This mixture was stable for at least a week at 4°C. For chronic studies, compound was prepared once a week and kept at 4°C in the dark. Methylprednisolone (MP) (Urbason^®^) used in the EBA experiments was purchased from Sanofi-Aventis (Frankfurt, Germany). For KLH immunization and MRL/lpr studies, MP succinate and prednisolone was purchased from Sigma. All corticoids were dissolved in the vehicle described above for each model. Phorbol 12-myristate 13-acetate (PMA; Sigma-Aldrich, Munich, Germany) and *N*-formyl-l-methionyl-l-leucyl-l-phenylalanine (fMLP; Sigma-Aldrich, Munich, Germany) were dissolved in Aqua ad injectable and PBS/2% ethanol, respectively.

### Generation of the vWFA2 Recombinant Protein and Anti-Murine vWFA2 IgG

Recombinant murine von Willebrand factor A-like domain 2 (vWFA2) of the NC1 of COL7 [aa 1048–1238 with five additional amino acids (GRAMG) at the N-terminus] were produced as previously described using prokaryotic expression (27). Rabbit anti-murine vWFA2 IgG was generated as previously described (28). IgG from rabbit serum was isolated using Protein G Sepharose Fast Flow affinity column chromatography (Amersham Biosciences, Freiburg, Germany) as described (28). Reactivity of IgG fractions was analyzed by IF microscopy on murine skin. Concentrations of the purified rabbit IgG were measured at 280 nm by spectrophotometer.

### Animal Models of EBA

For induction of experimental EBA by immunization in B6.s mice, we followed previously published protocols (11). Mice were monitored weekly for presence of skin lesions. If in an individual mouse, skin lesions affected 2% or more of the body surface area, it was randomized to one of the four treatment groups: (i) vehicle (0.5% methylcellulose, 0.1% Tween80 once daily by oral gavage), (ii) MP (20 mg/kg once daily by oral gavage), (iii/iv) LAS191954 at 1 or 3 mg/kg (once daily by oral gavage). Mice that did not reach this inclusion criterion 8 weeks after immunization were not considered further. Treatments were carried out for a total period of 6 weeks. Each week mice were clinically evaluated and the percentage of the affected body surface area determined by an investigator unaware of the treatment. At randomization and at the end of the treatment period, serum was obtained for determination of circulating antigen-specific IgG, which was performed as described (11). Compound plasma levels were determined by spectrometry as described (26) on samples obtained 1 h after the last compound administration. This time point corresponds approximately to the Cmax of the compound in plasma as determined in healthy mouse PK studies (data not shown). At the same time, ears of the mice were fixed in paraformaldehyde for later H&E staining for semiquantitative evaluation of the dermal leukocyte infiltration.

Induction of experimental EBA by antibody transfer in B6 mice was performed as described earlier (28). Same treatments as above were started one day before the first IgG injection and maintained throughout the 12-day experiment. Mice were clinically evaluated every fourth day, and the percentage of affected body surface area was noted.

### Assessment of T-Cell-Dependent Antibody Responses (TDAR) in Mice

Immunizations with KLH were performed as described (29) and the effect of compounds on the primary specific IgG subsequently assessed on day 15. Groups of six animals received LAS191954 (0.1, 0.3, 1, and 3 mg/kg) or MP (1, 3, and 10 mg/kg) or vehicle daily by oral gavage starting on the day of sensitization (day 1) until day 14. Terminal blood samples were collected 24 h after the last treatment administration (day 15) into EDTA tubes for anti-KLH antibody testing as described (29).

### MRL/lpr Studies

Baseline antibody measurements were determined at weeks 9–10 of age and used to randomize animals to experimental groups using QuickCalcs tool from GraphPad (http://www.graphpad.com/quickcalcs/randomize2/). Each group consisted of a minimum of eight animals. Animals were orally administered vehicle, LAS191954 or prednisolone. All test solutions were analyzed after each period of administration to recheck stability and compound concentration. On weeks 2 and 4, blood was sampled by submandibular bleeding for antibody level determination. Samples from all time points were analyzed for anti-dsDNA levels and anti-desmoglein 3 antibody levels. Antibodies to dsDNA and Dsg3 were determined using the Mouse anti-dsDNA total Ig ELISA Kit (Alpha Diagnostic Cat#5110) and the Mouse Desmoglein 3 Antibody ELISA kit (MBL International Cat#7597), respectively.

### ROS Release by Polymorph Nuclear Cells (PMN) and Monocytes

Polymorph nuclear cells are isolated from freshly drawn, heparin-anticoagulated blood from healthy volunteers by using PolymorphPrep™ (Axis-Shield GmbH, Heidelberg, Germany) according to the manufacturer’s protocol. Stock solutions of LAS191954 were prepared in DMSO at different concentrations starting at 3 × 10^−3^ M and following a fourfold bank dilution and stored at −80°C. For every experiment, LAS191954 was further diluted 1:250 in modified dye-free RPMI 1640 medium (Genaxxon, Ulm, Germany) containing 1% fetal calf serum and 25 mM HEPES to a fourfold assay concentration. MP was dissolved in modified dye-free RPMI 1640 medium containing 1% fetal calf serum and 25 mM HEPES to a fourfold assay concentration (2.0 × 10^−3^, 4.0 × 10^−4^, and 2.0 × 10^−4^ M). For release of ROS, PMA (10 ng/mL), fMLP (1 µM), and human recombinant C5a (100 nM; Hycultec, Beutelsbach, Germany) were prepared at a twofold assay concentration on white microwell plates (Greiner BioOne, Frickenhausen, Germany). For immune complex-induced activation, white microwell plates were coated with recombinant human COL7 (10 µg/mL), and after blocking with 1% bovine serum albumin (BSA), monoclonal anti-human COL7 IgG1 was added (2 µg/mL). Isolated PMN (4 × 10^6^ cells/mL) were diluted 1:2 with different concentrations (fourfold) of LAS191954 or (fourfold) of MP, and preincubated for 15 min at room temperature before added to the different stimuli on the assay plate. Untreated and DMSO-treated cells served as controls. By using the luminol (5-amino-2,3-dihydro-1,4-phthalazindione)-amplified chemiluminescence assay, released ROS was measured at a VICTOR™ 3 reader (PerkinElmer Inc., Waltham, MA, USA) over a period ranging from 60 to 180 min as described in detail elsewhere (30). For monocyte isolation, human peripheral blood mononuclear cells (PBMCs) were isolated from heparin-anticoagulated blood samples using the Ficoll density gradient (GE Healthcare, Freiburg, Germany) according to the manufacturer’s instructions. In a next step, human monocytes were purified from the isolated PBMCs with the magnetic cell separation (MACS) method using the monocyte isolation kit II human (Miltenyi, Bergisch- Gladbach, Germany) according to the manufacturer’s instructions. The purity of monocytes and neutrophils was evaluated by fluorescent staining with PE/Cy7 anti- human CD14 antibody (clone HCD14, Biolegend, Kobenz, Germany) and FITC anti-human CD16 antibody (BD Biosciences, Heidelberg, Germany) in a flow cytometer measurement (MACSQuant^®^ Analyzer 10, Miltenyi). ROS release assay using monocytes was performed as described above.

### *Ex Vivo* Dermal–Epidermal Separation (DES) Assay

Cryosections of human skin were incubated with rabbit anti-human COL7^FNIII8-FNIII9^, followed by the addition of human PMN as described (31). To evaluate the potential effect of LAS191954, the compound or vehicle (0.1% DMSO) was added at varying concentrations along with the PMN onto the skin sections. Separation of the dermal–epidermal junction was calculated as length of separation divided by total length of the dermal–epidermal junction on skin section measured with BZ-9000 fluorescence microscope (Keyence, Frankfurt, Germany). Evaluation was conducted by an investigator unaware of the section’s treatments.

### Chemotaxis of PMN

Polymorph nuclear cells were prepared from citrated blood of healthy donors by a combination of sedimentation and Ficoll density gradient centrifugation as described (10). Sedimented cells were incubated with ice-cold aqua dest for lysis of erythrocytes, washed in ice-cold phosphate buffered saline (PBS), and suspended in PBS at 4 × 10^6^ cells/mL. To determine the effect of the PI3Kδ inhibitor LAS191954 on neutrophilic chemotaxis, stock solutions of the inhibitor stored at −80°C (prepared in DMSO at different concentrations starting at 3 × 10^−3^ M and following a fourfold bank dilution) were further diluted 1:500 in PBS containing 1% BSA to a twofold assay concentration and tested in a slightly modified chemotaxis assay (32). In brief, interleukine (IL)-8 (6 nM; Peprotech, Hamburg, Germany), fMLP (10 nM), and human recombinant C5a (12 nM) were diluted in PBS with Ca^2+^, Mg^2+^/0.1% BSA and added to the bottom wells of the chamber. After covering the bottom wells with a polycarbonate membrane (pore size: 5 µm, Costar Nucleopore GmbH, Tübingen, Germany), the top wells were filled with 1 × 10^5^ freshly prepared neutrophils in PBS with Ca^2+^, Mg^2+^/1% BSA that were preincubated with different concentrations of the PI3Kδ inhibitor LAS191954 (10 min, 37°C). After incubation for 1 h at 37°C, migration was terminated by removing the cells from the top wells. Migrated cells were transferred from the bottom wells to a microtiter plate and lysed with 0.1% hexabromide solution. Number of migrated neutrophils was determined *via* endogenous myeloperoxidase activity by adding myeloperoxidase substrate tetramethylbenzidine (Life Technologies, Paisley, UK). The redox reaction was stopped by 0.9 M sulfuric acid and oxidized tetramethylbenzidine measured at λ = 450 nm. The number of migrated neutrophils was calculated from a standard curve of cell lysates run in parallel.

### Statistical Analysis

Statistical analysis was performed using SigmaPlot 13.0. Used tests are indicated at each figure legend. A *p*-value of <0.05 was considered significant. If not otherwise indicated, mean and SEM are presented.

## Results

### LAS191954 Ameliorates Already Established Disease in Immunization-Induced EBA

LAS191954 is a specific and potent inhibitor of the p110δ catalytic isoform of PI3K with a reported enzymatic potency of 2.6 nM (26). The compound is 30-fold selective versus the p110β (IC50 94.2 nM) and p110γ (IC50 71.7 nM) isoforms and 3,000-fold over the p110α isoform (IC50 8226 nM). A similar selectivity profile as in the enzymatic assays has been observed in PI3Kδ-dependent (human anti-IgD activated PBMC) or PI3Kβ-dependent (S1P-activated HUVEC) cellular assays, with reported IC50 of 4.6 nM and 295 nM, respectively. No off-target activity has been reported in a panel of GPCRs, transporter, and kinases up to 10 µM. *In vivo*, LAS191954 shows excellent oral bioavailability in preclinical species and demonstrates pharmacological activity in both acute and chronic settings (26).

**Figure 1:**
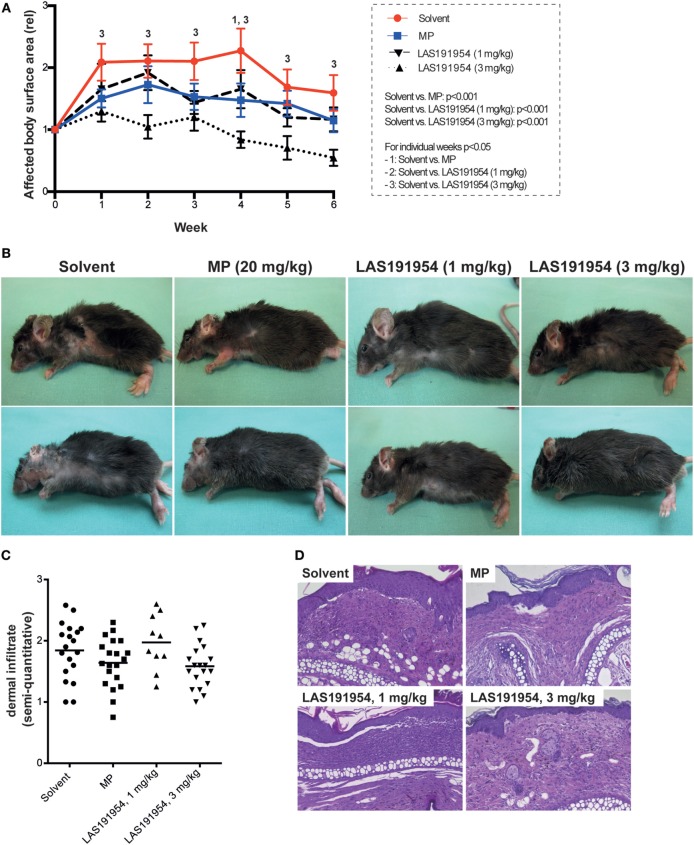
Pharmacological PI3Kδ inhibition improves already clinically manifested experimental Epidermolysis bullosa acquisita (EBA). B6.s mice were immunized with COL7 for induction of experimental EBA. When skin lesions affected 2% or more of the body surface area, individual mice were randomly allocated to one of the four treatment groups. **(A)** Clinical disease severity, expressed as affected body surface area in relation to the time of allocation to treatment (week 0). Data are based on 11 mice per group, with the exception of LAS191954 1 mg/kg [*n* = 6; two-way ANOVA (taking time and treatment as variables) with Holm-Sidak posttest]. The global *p* values of testing solvent versus the treatments is given in the box on the left. For the posttest, *p* values < 0.05 are indicated by numbers, whereas “1” indicates a difference between solvent and methylprednisolone (MP), “2” a difference between solvent and LAS191954 (1 mg/kg), and “3” a difference between solvent and LAS191954 (3 mg/kg). **(B)** Representative clinical images of two mice from each group taken at the end of the experiment. **(C)** Semiquantitative evaluation of the dermal infiltrate of the ears at week 6 of the experiment, with 0 indicating no, 1 mild, 2 moderate, and 3 severe infiltration. While a tendency toward lower infiltration scores was noted in MP and LAS191954 (3 mg/kg), this was not statistically significant. Each datapoint represents one ear per group (if possible, both ears were evaluated). Statistical significance was calculated using ANOVA. **(D)** Representative H&E-stained sections from all treatment groups at week 6 of treatment. Original magnification 200×.

We took advantage of the above selective and potent compound profile to first evaluate the therapeutic effect of pharmacological PI3Kδ inhibition in EBA, by treating mice with active disease with two different doses of LAS191954 daily for 6 weeks. Allocation to treatment was performed when skin lesions affected 2% or more of the body surface area in individual mice. To rule out a potential allocation bias, the time after immunization and the affected body surface area were analyzed in each group. Both, time after immunization (Figure S1A in Supplementary Material) and affected body surface area at time of allocation (Figure S1B in Supplementary Material) showed no significant differences in all groups. In vehicle-treated animals, affected body surface area increased twofold within the first week, remained constant until week 4, and then gradually decreased until the end of the 6-week treatment period. As shown in Figures 1A,B, in mice treated with LAS191954 at 1 mg/kg, disease progression was impaired, without a statistical significant difference between vehicle and the LAS191954-treated (1 mg/kg) group during individual time points of the 6-week observation period. Of note, a higher dose of LAS191954 (3 mg/kg) completely abolished disease progression and even improved disease (defined as less body surface area affected by skin lesions) starting from week 4. In line with the dose-dependent clinical effects, compound plasma exposures increased proportionally with the dose in the treated animals as assessed at the 1 h post-administration time point (corresponding to the Cmax) on the last day of the study. Free plasma levels were 575 ± 245 nM for the 3 mg/kg- and 135 ± 71 nM for the 1 mg/kg-treated group. These results were in agreement with oral pharmacokinetic studies performed in healthy mice (data not shown). High-dose corticosteroid treatment (20 mg/kg MP) was used as a reference treatment in a head-to-head comparison, because previous work demonstrated beneficial effects of high-dose MP treatment in this model (33). In line with previous data (where MP was given i.p.), orally administered MP hindered disease progression (with significant differences observed at week 4), but failed to improve skin blistering (Figures 1A,B). Investigation of the dermal leukocyte infiltrate, evaluated in ears of mice at the end of the treatment period, did not show any differences among the groups (Figures 1C,D).

### LAS191954 Does Not Modify Circulating Autoantibody Titer in Experimental EBA

**Figure 2:**
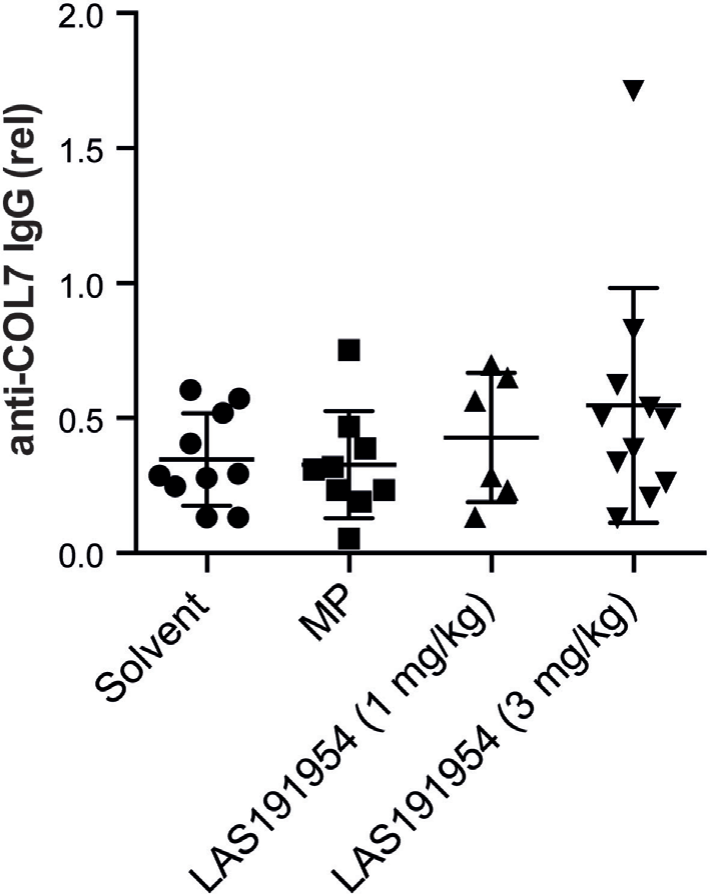
Circulating anti-COL7 IgG remains unaltered after treatment with either LAS191954 or methylprednisolone. At randomization (week 0) and at the end of the treatment (week 6), serum was obtained from selected mice. The graph shows the mean and STW of the relative serum concentration of anti-COL7 IgG antibodies at week 6 in relation to week 0. In all groups, a decrease of COL7-specifig IgG was noted, with no statistically difference observed among the groups. Data are based on 6–11 mice per group. Each dot represents the data from one animal. Statistical analysis was performed using one-way ANOVA.

In order to ascertain whether the mechanism of action of LAS191954 in experimental EBA involves the antibody response, we first assessed whether chronic treatment with the compound had an effect on circulating autoantibody concentrations. To address this, we determined the levels of anti-COL7 specific IgG in mice treated for 6 weeks with LAS191954 at the end of the treatment period (same experiment as in Figure 1). Of note, a similar decrease of COL7-specific IgG was observed in all treatment groups (Figure 2), suggesting that LAS191954 had no impact on autoantibody levels over this time period in this model.

This result was in contrast with the reported role of PI3Kδ in TDAR and B cell function (20). To further study the effect of LAS191954 on humoral responses, we checked whether LAS191954 could prevent antibody responses to a model immunogen like KLH in CD1 mice. In this setting, the response is elicited in the absence of potent adjuvants, unlike in the EBA model. Indeed, LAS191954 dose-dependently inhibited the primary IgG response with a maximal response attained at 0.3 mg/kg (Figure S2 in Supplementary Material). We further tested whether LAS191954 could reduce the spontaneous production of autoantibodies in MRL/lpr mice, a mouse strain showing an autoimmune lymphoproliferative disorder with autoantibodies to various antigens including the skin-specific antigen, desmoglein (Dsg) 3 (34). Chronic treatment of mice daily for 4 weeks progressively and dose-dependently decreased the titer of circulating dsDNA-specific and Dsg3-specific autoantibodies (Figure S3 in Supplementary Material) with the dose of 1 mg/kg being maximal and similar to the effect of prednisolone in this model.

Taken together, these results indicate that the observed therapeutic effect of LAS191954 in immunization-induced EBA seems to be independent of modulatory effects on antibody responses, despite LAS191954 being able to modulate antibody responses in other induced and spontaneous mouse models at similar or lower doses, suggesting that differences in the way the immune response is elicited in all models may account for the results observed.

### LAS191954 Prevents Onset of Inflammation in Antibody Transfer-Induced EBA

**Figure 3:**
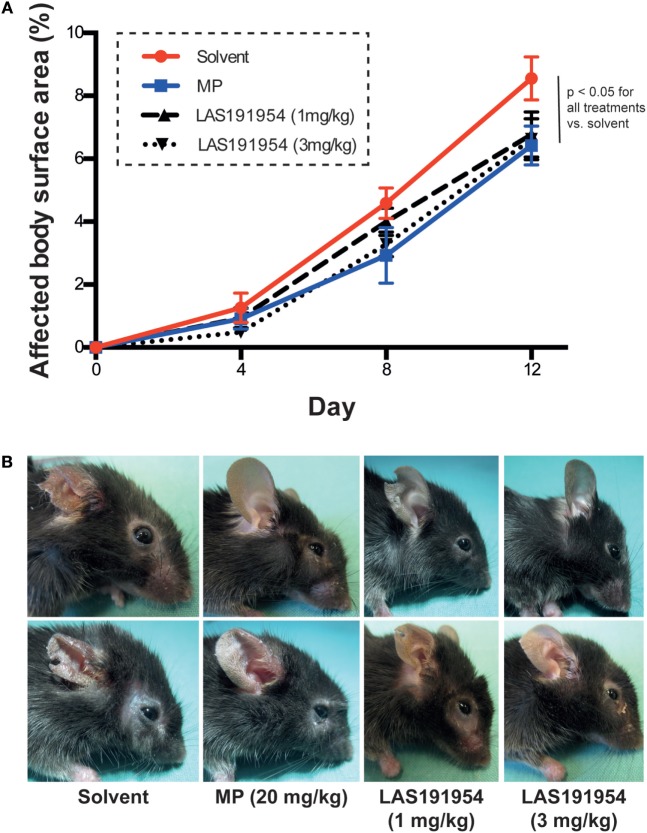
LAS191954 treatment prevents the onset of antibody transfer-induced Epidermolysis bullosa acquisita (EBA). Experimental EBA was induced in B6 mice by repetitive injections of anti-COL7 IgG. Simultaneously, mice were treated with the indicated compounds. **(A)** In all groups, experimental EBA was induced. Data are based on 10 mice per group. Statistical analysis was performed using two-way ANOVA (taking time and treatment as variables) with Holm-Sidak posttest. **(B)** Representative clinical images of two mice from each group taken at the end of the experiment.

We next evaluated the effect of LAS191954 on antibody transfer-induced EBA. This model recapitulates the effector phase of the disease, as it is induced by direct transfer of anti-COL7 IgG and requires myeloid cell activation through activating FcγR (9). The same doses of LAS191954 and MP as in the immunization-induced EBA model above were administered to groups of mice following a preventive scheme as described in Section “Materials and Methods.” The results show that small but significant effects of both MP and LAS191954 were obtained at reducing blistering versus vehicle-treated mice (Figure 3) suggesting a potential effect of the compound in the effector phase.

Taken together, the above results indicate that LAS191954 has a pharmacological effect in two different EBA mouse models at similar effective doses and suggest that inhibition of pathogenic myeloid cell activation rather than blocking autoantibody production accounts for the observed therapeutic effect.

### LAS191954 Inhibits Myeloid Cell Function *In Vitro*

**Figure 4:**
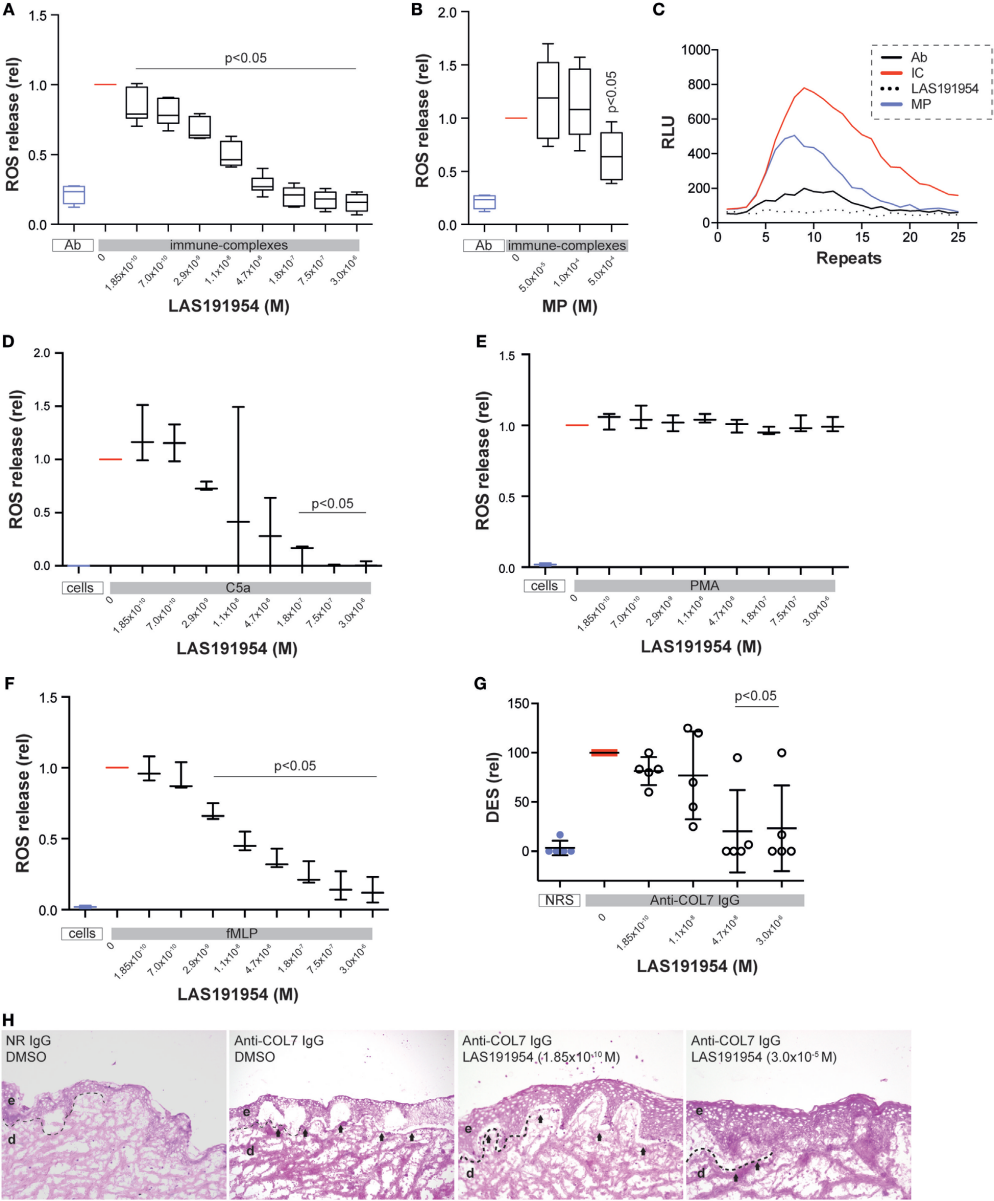
PI3Kδ is predominantly required for immune complex-induced ROS release from human polymorph nuclear cells (PMN). **(A–C)** Human PMNs were activated using immune complexes and their activation was determined by measuring ROS release over time in **(A)** the absence or presence of LAS191954. The graph shows the ROS release in relation to the vehicle control (*n* = 6/group). **(B)** In the same experimental setting, methylprednisolone was used as a reference treatment (*n* = 6/group). **(C)** Example of ROS release. The *y*-axis shows the relative light units, which correspond to the ROS release. Repeats indicate the time; one repeat approximately corresponds to 2.4 min. Human PMN were activated by **(D)** C5a, **(E)** PMA, and **(F)** fMLP, respectively, in absence or presence of LAS191954. All data in graphs **(D–F)** is based on three experiments per group. **(G)** Cryosections of human skin were incubated with rabbit anti-human COL7 and human PMN from healthy donors. This leads to dermal–epidermal separation (DES), which is shown in relation to vehicle-treated sections. Data are based on five experiments per group. **(H)** Representative, H&E-stained sections of human skin sections incubated with anti-human COL7 and human PMN from healthy donors. For all panels, one-way ANOVA with Holm-Sidak posttest was used for statistical analysis. Abbreviations: e, epidermis; d, dermis, arrows indicate DES, dotted line indicates location of dermal–epidermal junction.

To validate these assumptions, we next set out to determine whether the mechanism of action of LAS191954 may be driven by effects on myeloid cells. For this, we isolated PMN from human volunteers’ peripheral blood and assessed the effect of LAS191954 on the release of ROS induced by immune complexes. LAS191954 dose-dependently reduced the immune complex-induced ROS release from human PMN with an IC50 of 11 nM (Figure 4A). These findings were validated by use of another PI3Kδ-selective inhibitor (IC-87114) (23), which also dose-dependently and well within the reported IC50, reduced the immune complex-induced ROS release from human PMN (not shown). Furthermore, we investigated the impact of LAS191954 on immune complex-induced ROS release from human monocytes, which recently has been shown to contribute to EBA pathogenesis (13). Like in PMN, LAS191954 also impaired the immune complex-induced ROS release from human monocytes (Figure S4 in Supplementary Material). The effect of MP on immune complex-induced ROS release was also investigated in parallel. As reported earlier, when MP was given at the same dose (35), MP impaired the immune complex-induced ROS release from PMN. Yet, and in contrast to LAS191954, higher concentrations were required, consistent with the high doses that are used *in vivo* (Figures 4B,C). To test if also other known inducers of PMN activation are sensitive to LAS191954 treatment, PMN were activated using PMA, fMLP, or C5a. While LAS191954 had no impact on PMA-induced ROS release (Figure 4E), C5a-induced and fMLP-induced ROS release was dose-dependently inhibited with IC50s of 19 and 7.6 nM, respectively (Figures 4D,F). Based on the potency and selectivity profile described for the compound [see above and Ref. (26)], these results suggest that inhibition of PI3Kδ is the mechanism that accounts for the observed inhibition of ROS release.

In order to evaluate if LAS191954 can prevent a detrimental downstream effect of ROS on skin, we made use of a ROS-dependent *in vitro* model of human EBA. This model emulates the EBA prototypical DES on cryosections of human skin co-incubated with human COL7 antibodies bound to human PMN, reflecting IC-induced PMN activation (12). We measured the epidermolytic effect in the presence of different compound concentrations. In line with the previous results, LAS191954 dose-dependently and almost completely abolished *ex vivo* dermal-epidermal separation at and above concentrations of 47 nM (Figures 4G,H), suggesting that this process was also dependent on PI3Kδ activation.

**Figure 5:**
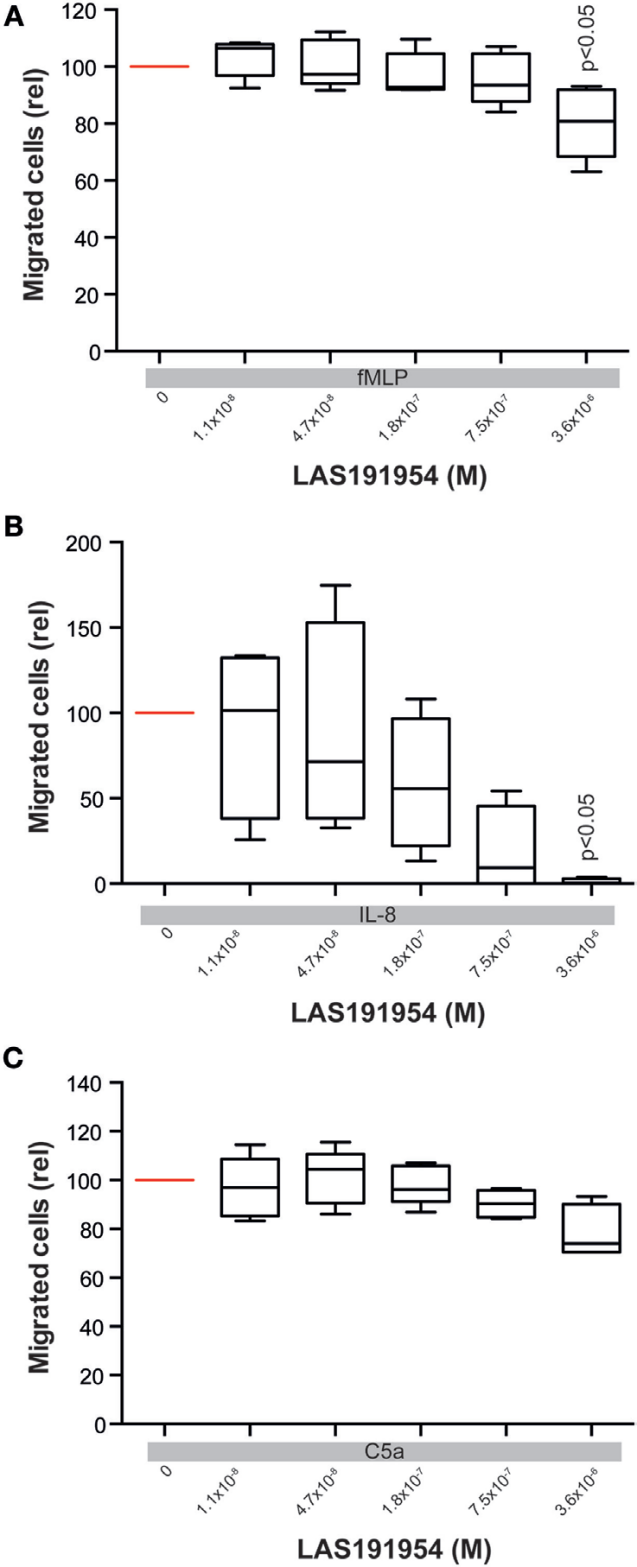
Blockade of PI3Kδ mainly blocks IL-8 induced polymorph nuclear cells (PMN) migration. Migration of human PMN was induced using **(A)** fMLP, **(B)** IL-8, or **(C)** C5a in the presence of LAS191954. All panels show migrated cells in relation to the positive control (vehicle, no LAS191954). Data are based on five experiments per group, with the exception of C5a-induced migration (*n* = 4). For all panels, one-way ANOVA with Holm-Sidak posttest was used for statistical analysis.

In experimental EBA, the crucial CD18-dependent myeloid extravasation into the skin is partially driven by IL-8 mouse homolog cytokines and the complement cascade component, C5a (36, 37). We, therefore, next evaluated the effect of LAS191954 on human PMN migration induced by fMLP, IL-8, or C5a. Compared to the fMLP-induced ROS release, the impact of LAS191954 on fMLP-induced myeloid cell migration was minimal, albeit significant at the highest tested dose (Figure 5A). Similarly, C5a-induced migration was unaffected (Figure 5C). In contrast, LAS191954 completely abolished the IL-8 induced migration of human PMN with an IC50 of 93 nM (Figure 5B). Altogether, these results demonstrate that LAS191954 can efficiently modulate distinct human myeloid cell functions.

## Discussion

The aim of this study was to test whether pharmacological inhibition of PI3Kδ with a novel selective inhibitor can modulate the progression of experimental EBA in mice. We demonstrate that chronic administration of this compound using a therapeutic scheme (i.e., in established clinical disease induced by COL7 immunization) improves and even normalizes the cutaneous clinical manifestations in a dose-dependent fashion. This effect is superior to that obtained with a high dose of corticosteroid and does not seem to be mediated by modulation of antibody responses. Furthermore, in an antibody transfer-induced model of EBA reproducing the effector phase of the disease, the compound can prevent the blistering induced by pathogenic antibody transfer in a similar way as corticosteroids.

Myeloid cells are essential for experimental EBA development in mice and distinct contributions of different PI3K isoforms to neutrophil function have been described. In this regard, previous studies have shown that loss of PI3Kβ expression conferred a substantial, but incomplete, protection from inflammation in antibody transfer-induced EBA. Furthermore, chimeric mice created by adoptive transfer of PI3Kβ-deficient bone marrow cells into irradiated wild-type mice were similarly protected from EBA induction. Further *in vitro* experiments indicated that PI3Kβ and PI3Kδ may act synergistically to release ROS from immune complex-activated murine and human myeloid cells (17, 38). Similarly, combined pharmacological inhibition or genetic deficiency of PI3Kβ and PI3Kδ were found to be necessary to inhibit the mouse neutrophil ROS response to *Aspergillus fumigatus* hyphae (39). Hence, both, PI3Kβ and δ seem to be the driving forces for immune complex and pathogen-driven ROS release in neutrophils.

The differential potency of our compound in each PI3K isoform allows us to further assess the contribution of each isoform in the biological process studied. *In vitro*, the compound inhibits ROS release from human PMN stimulated with immune complexes or other physiologically relevant inducers (fMLP, C5a) and does so at concentrations expecting to inhibit mainly the PI3Kδ isoform while sparing the other class I PI3K isoforms. In addition, the epidermal–dermal separation promoted by immune complex-induced ROS release also occurs at nanomolar concentrations at which the δ isoform is predominantly inhibited. Conversely, several neutrophil responses were insensitive (i.e., C5a-induced PMN migration) to the compound or only at very high concentrations (i.e., fMLP-induced PMN migration) indicating that either no PI3K isoform is involved or only the α isoform is playing a role in those pathways, in agreement with previous reports (40, 41). We further corroborated these findings with IC-87114, another highly PI3Kδ inhibitor that is >50 times selective over PI3Kβ (23). IC87114 dose-dependently reduced the immune complex-induced ROS release from human PMN at concentrations exclusively covering the PI3Kδ (not shown). Considering these results along with our findings of an unaltered dermal neutrophil infiltrate at the end of the LAS191954 treatment (Figures 1C,D), this indicates that the PI3Kδ is predominantly required for ROS release from PMN, while having less pronounced effects on PMN migration.

When analyzing the *in vivo* situation, the calculated unbound compound concentrations in plasma (575 ± 245 nM) for the dose at which the compound exerts its maximum efficacy (3 mg/kg) in the experimental EBA model do not allow to attribute the observed effects solely to the inhibition of PI3Kδ. Whereas this dose may have ensured an extended inhibition period for PI3Kδ, partial and transient coverage of PI3Kβ and γ may also have occurred during the dosing period, suggesting that inhibition of more than one isoform may be necessary for full efficacy in the EBA models. This hypothesis is aligned with the reported PI3Kδ and PI3Kβ essential roles in the IC-induced neutrophil response. In addition, a predominant use of PI3Kβ by mouse neutrophils and of PI3Kδ by human neutrophils for the production of ROS in response to immune complexes was reported (41), indicating that species-specific differences in the usage of distinct PI3K isoforms in mouse and human neutrophils may be possible. However, an additional, highly selective PI3Kδ inhibitor, IC-87114, also impaired the induction of antibody transfer-induced EBA (data not shown), thus further supporting PI3Kδ as a key PI3K isoform in this model. This would be in agreement with our *in vivo* observations and also with our results in human PMN where PI3Kδ seems to be the only isoform required for the functions studied. Furthermore, our finding of an unaltered dermal neutrophil infiltrate at the end of the treatment with LAS191954 in the immunization-induced EBA model indicates that the PI3Kδ isoform is not essential for PMN migration while being required for IC-activated ROS release.

We found that the autoantibody response to COL7 was not altered by the chronic administration of the compound as determined by IgG levels on the last day of the experimental EBA study, ruling out the potential modulation of the adaptive response as a contributor to the therapeutic effect. Based on the biology of the target, this result was unexpected and is in contrast with the compound’s observed inhibitory effect on the antibody response to T-cell-dependent antigens and on the autoantibody titer in a spontaneous autoimmunity mouse strain (MRL/lpr) at doses similar or below the ones used in the EBA study. Although not a clear explanation can be put forward for this discrepancy, a potential difference may lie in the way the antibody response is elicited in the EBA model with the use of a very potent adjuvant and inducer of IgG production (unpublished observations). Alternatively, it is possible that effects of PI3Kδ inhibition on B cells may not have become evident during the 6-week observation period because of the 7-week autoreactive plasma cells half-life in this model (42). In addition, as we observed a decrease of anti-COL7 IgG in all groups, including the control group, the potential effects of PI3Kδ inhibition could have been masked. Finally, the particular T cell-dependent autoantibody generation in immunization-induced EBA (11) may be insensitive to pharmacological PI3Kδ inhibition.

Taken together, our results provide new evidence that targeting the PI3Kδ pathway may be a suitable approach for the treatment of EBA, and possibly also other pemphigoid diseases such as bullous pemphigoid in which activation of myeloid cells has a key pathogenic role.

## Ethics Statement

Foreskin and blood collections from healthy volunteers were performed after written informed consent was obtained. All experiments with human samples were approved by the ethical committee of the Medical Faculty of the University of Lübeck and were performed in accordance with the Declaration of Helsinki. *Laboratory animals*: C57Bl/6 (B6) and B6.SJL-H2s (B6.s) mice were obtained from colonies held at the animal facility at the University of Lübeck. Mice were housed under specific pathogen-free conditions and provided standard mouse chow and acidified drinking water *ad libitum*. Mice aged 6–10 weeks were used for the experiments. All clinical examinations, biopsies, and bleedings were performed under anesthesia with i.p. administration of a mixture of ketamine (100 μg/g) and xylazine (15 μg/g). Evaluation of skin lesions was performed as described (10). Animal experiments were approved by local authorities of the Animal Care and Use Committee (Kiel, Germany) and performed by certified personnel. For KLH immunization studies, male Crl:CD1 (ICR) mice were purchased from Charles River. Female MRL/MpJ-Faslpr/J mice were purchased from The Jackson Laboratory (Bar Harbor, ME, USA). Animals were housed in polycarbonate cages, with free access to water and non-purified stock diet and allowed to condition for 2 weeks in their new environment at 22 ± 2°C, 40–70% relative humidity and 12 h:12 h light:dark cycles. All animal care and experimental procedures followed the European Community Directive 86/609/CEE and the Autonomous Catalan law (Decret 214/ 1997) for the use of laboratory animals and were approved by the Almirall Animal Experimentation Ethical Committee.

## Author Contributions

HK, AK, NG, RL, MP, MA, KB, and CB performed experiments. DZ, RL, and CB analyzed the data and wrote the manuscript. NG, RL, and CB designed the study. All authors read, commented, and approved the final version of the manuscript.

## Conflict of Interest Statement

RL, MA, MP, NG, and CB are employees of Almirall S.A. Costs for this research were in part covered by a research agreement between Almirall and the University of Lübeck. The remaining authors declare that the research was conducted in the absence of any commercial or financial relationships that could be construed as a potential conflict of interest.
