# Supplementary figures and images for: Therapeutic Effect of a Novel Phosphatidylinositol-3-Kinase δ Inhibitor in Experimental Epidermolysis Bullosa Acquisita
Source: Front Immunol. 2018 Jul 12;9:1558. doi: 10.3389/fimmu.2018.01558 (PMC6052048; doi:10.3389/fimmu.2018.01558)

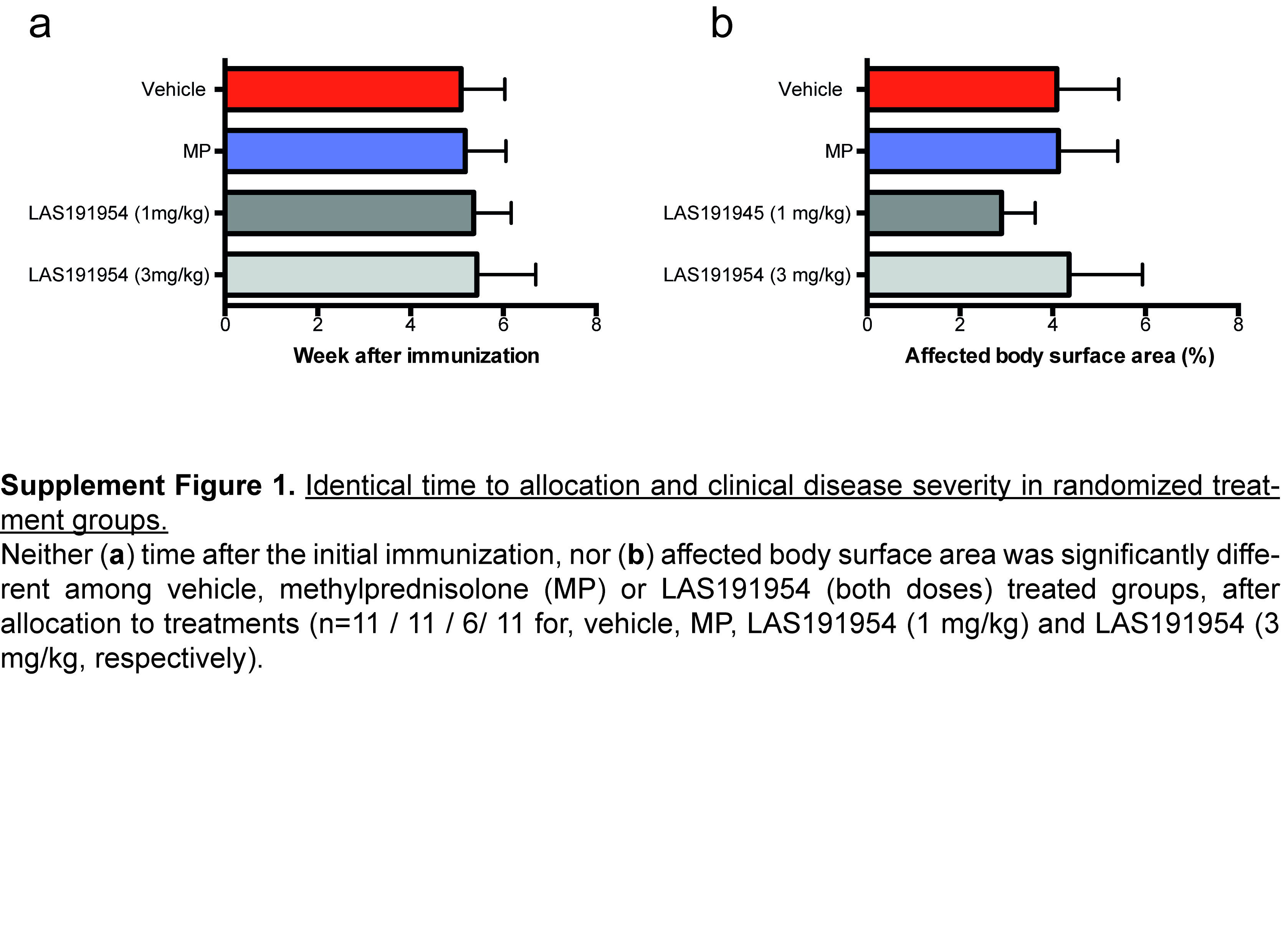

Supplement: Supplementary file 1 [file image_1.TIF]

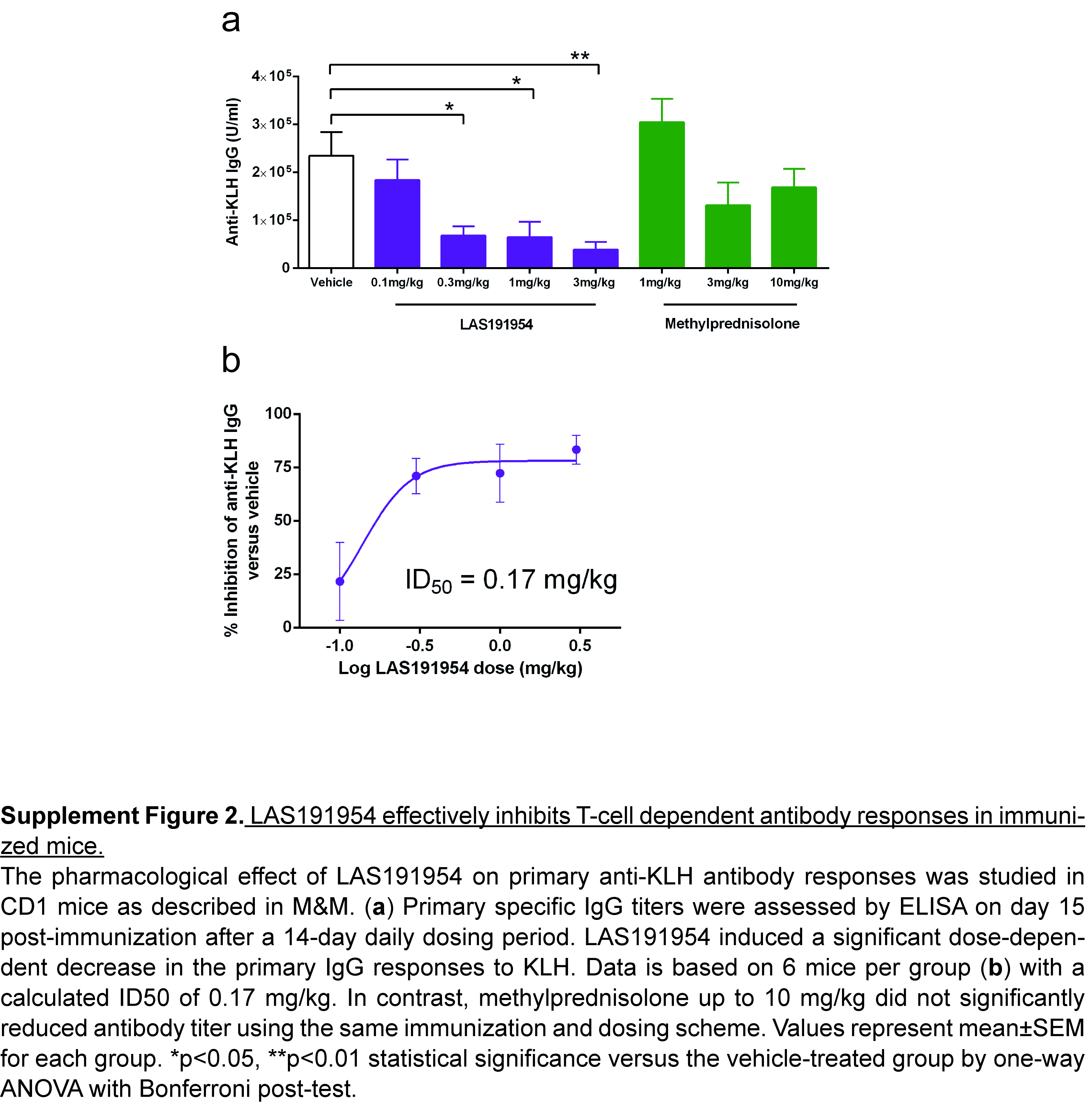

Supplement: Supplementary file 2 [file image_2.TIF]

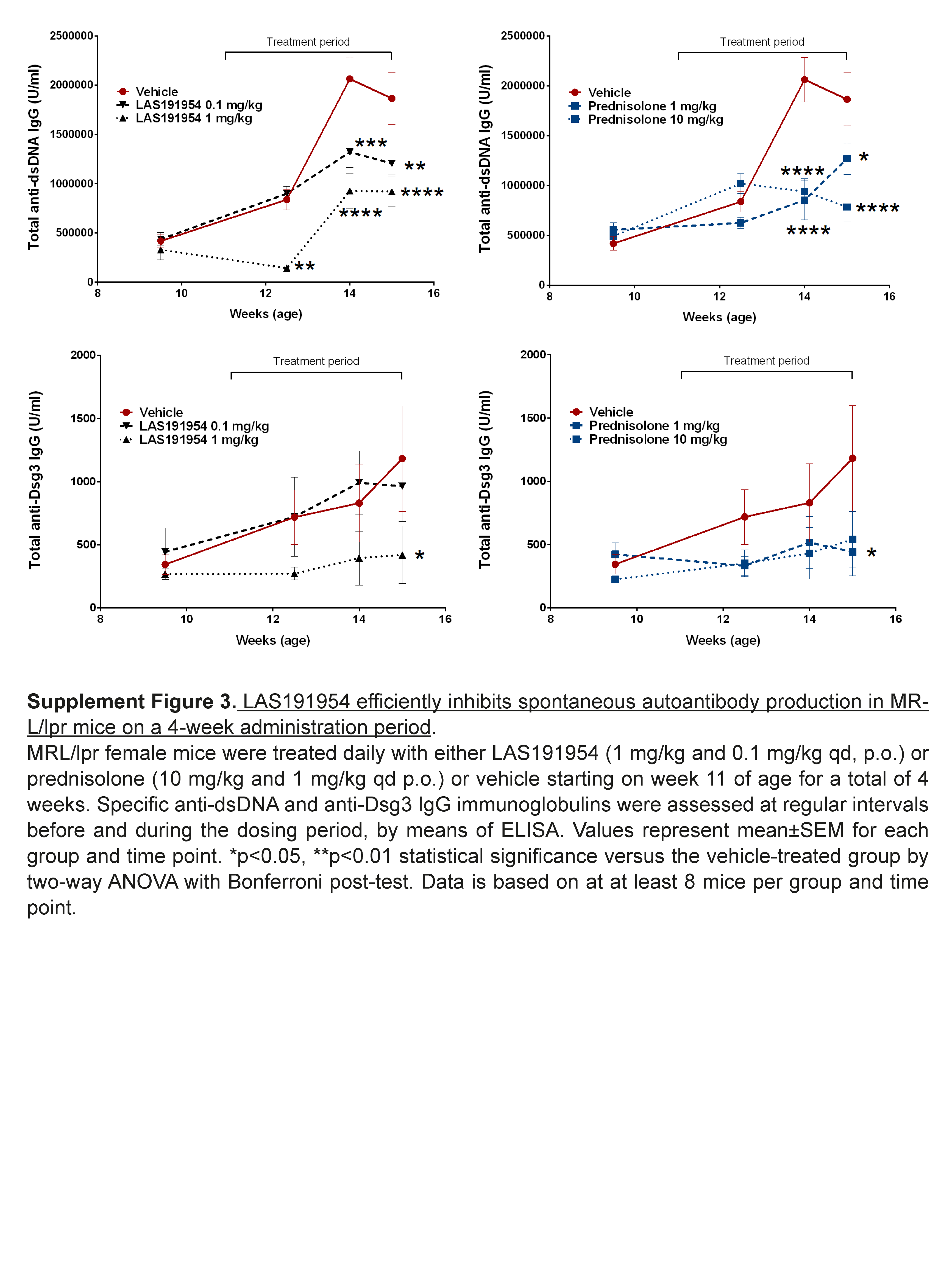

Supplement: Supplementary file 3 [file image_3.TIF]

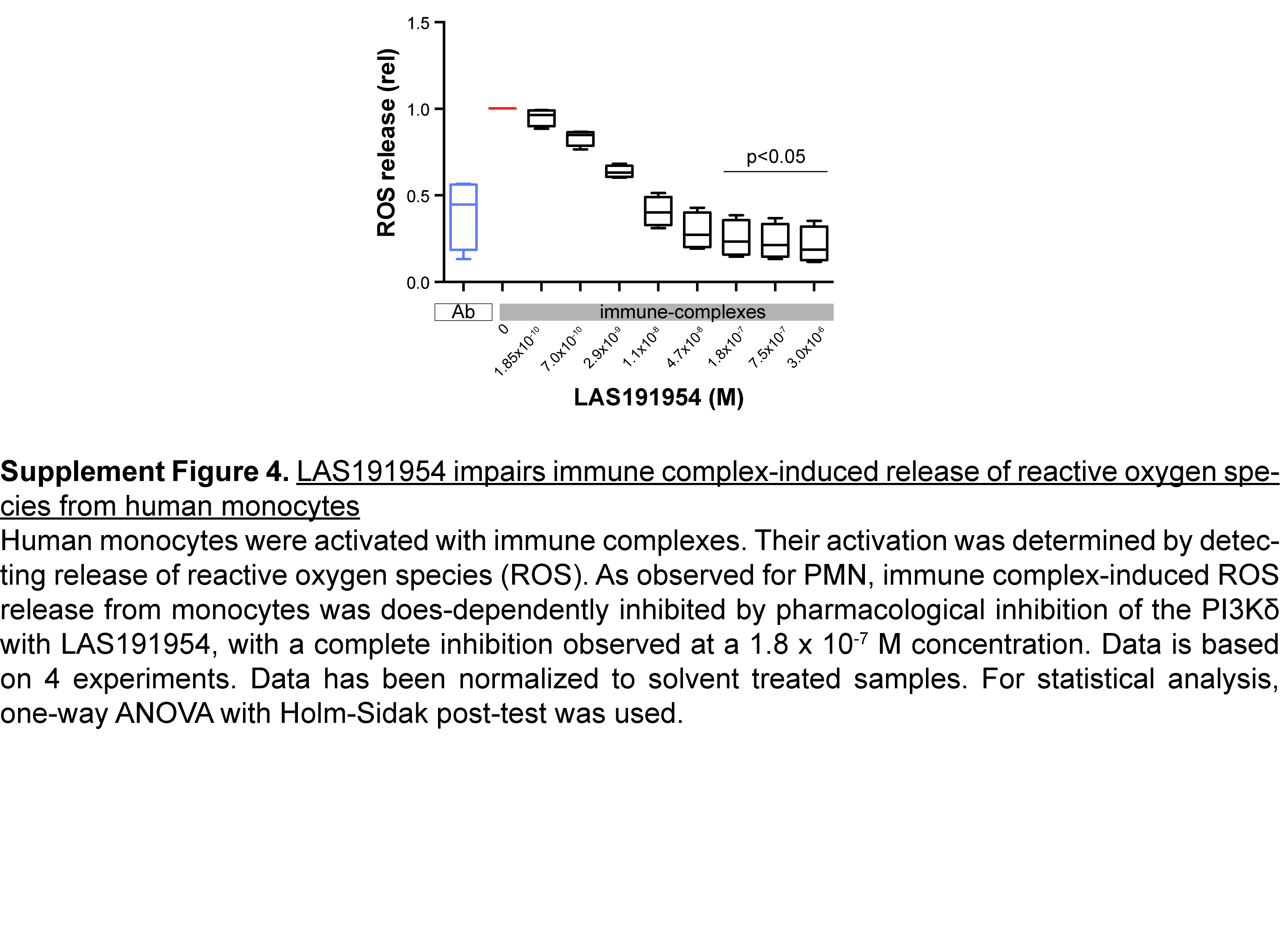

Supplement: Supplementary file 4 [file image_4.TIF]
